# Bone turnover markers to assess jawbone quality prior to dental implant treatment: a case-control study

**DOI:** 10.1186/s40729-020-00264-0

**Published:** 2020-11-03

**Authors:** Keisuke Yasuda, Shinsuke Okada, Yohei Okazaki, Kyou Hiasa, Kazuhiro Tsuga, Yasuhiko Abe

**Affiliations:** Department of Advanced Prosthodontics, Graduate School of Biomedical and Health Sciences, Hiroshima University, 1-2-3, Kasumi, Minami-ku, Hiroshima, 734-8553 Japan

**Keywords:** Bone implant interactions, Clinical research, Prosthodontics, Radiology, Imaging

## Abstract

**Background:**

Bone quality is as important as bone mineral density in terms of bone strength. Bone turnover markers (BTMs) are clinical indicators of bone quality. In implant dentistry, bone quality is considered equivalent to bone density on radiographic assessments. The purpose of this study was to determine whether the BTM values are reflected in jawbone condition by evaluating the relationship at baseline and during follow-up in patients with prosthodontic implants.

Computed tomography (CT) scans were obtained and BTM (osteocalcin, bone-specific alkaline phosphatase, pyridinoline cross-linked carboxyterminal telopeptide of type I collagen, and crosslinked N-telopeptide of type I collagen) levels in blood samples were measured in partially edentulous eighteen patients before implant surgery. During the follow-up observation after implant surgery, marginal bone loss (MBL) was measured on dental radiography. We investigated the relationship between the presence of BTM abnormalities and radiographic bone density.

**Results:**

More women than men had abnormal BTM values. Bone turnover was accelerated in the group of women with abnormal BTM values. The density of cancellous bone at the implant placement site was significantly lower in the patients with abnormally high BTM values than in their counterparts with BTM values in the normal range.

**Conclusions:**

Female patients who undergo implant treatments may have reduced bone quality; evaluations of bone strength will require assessments of both BTMs and the density of cancellous bone.

## Background

Dental implants have become an established prosthodontic treatment for missing teeth, with survival rates now exceeding 97% [1, 2]. The most popular current method of bone quality assessment is that developed by Lekholm and Zarb, who introduced a scale that ranges from 1–4 and is based on a radiographic assessment and the sensation of resistance experienced by the surgeon when preparing the implant site [3]. However, the initial studies excluded patients with type IV bone (“soft” bone), in whom implant treatment was considered more likely to fail earlier [3].

Recent review has reported that implants placed in patients with systemic osteoporosis did not present higher failure rates than those placed in patients without osteoporosis [4]. Furthermore, there are no data that contraindicate the use of dental implants in patients with osteoporosis [5], even though a correlation has been found between skeletal bone density and jawbone density [6].

In orthopedic surgery, osteoporosis is characterized by compromised bone strength that predisposes a patient to an increased risk of fracture [7]; this suggests that both bone quality and bone mineral density (BMD) contribute to the risk of fracture. Bone quality, defined as “the sum of all characteristics of bone that influence the bone’s resistance to fracture” [8], is independent of BMD. Bone quality is determined by the characteristics of the bone matrix, including the microarchitecture, bone turnover, accumulation of microdamage, degree of calcification, and collagen content [9, 10]. Many clinical studies have indicated that the increase in BMD following treatment with anti-resorptive drugs does not reflect the proportional reduction in relative fracture risk [11], which suggests that bone quality plays an important role in bone strength. In a consensus statement issued by the National Institutes of Health, bone quality was defined more specifically by bone architecture, bone turnover, bone mineralization, and the accumulation of microdamage [7].

Research on bone turnover markers (BTMs) has increased considerably over the past decade. The use of BTMs as a method of biochemically monitoring bone metabolism requires measurement of the enzymes and proteins released during bone formation and measurement of the degradation products produced during bone resorption. Various biochemical markers are now available that allow specific and sensitive assessments of the rates of skeletal bone formation and resorption [12]. Previous studies reported significant declines in areal BMD measured by DXA of the total hip and ultradistal radius [13] and high levels of both formative and resorptive markers were associated with reduced BMD measured by CT at the total hip, but not at the lumbar spine [14].

However, in implant dentistry, bone quality is still considered to be equivalent to radiographically assessed bone density [3, 15]. The paradigm of bone quality has shifted from density-based assessments to structural evaluations of bone; however, clarifying bone quality from structural evaluations has remained challenging in implant dentistry because devices suitable for accurate evaluations of bone structure have yet to be developed. Therefore, we focused on BTM values as a clinical indicator of bone quality and hypothesized that patients with abnormal BTM value may have an abnormal jawbone quality, which may affect the prognosis of the implant.

The purpose of this study was to determine whether the BTM values are reflected in jawbone condition by evaluating the relationship at baseline and during follow-up in patients with prosthodontic implants.

## Materials and methods

### Patient selection

Patients treated at Hiroshima University Hospital were included in the study if they remained partially edentulous in the planning area for at least 3 months, if one or two splinting fixed prostheses in the mandible posterior area were planned, if they had never been treated for osteoporosis, and if they had no subjective or objective symptoms of osteoporosis, such as back pain caused by a fractured or collapsed vertebra, loss of height over time, or stooped posture. Eighteen patients (11 women, 7 men) fulfilled the inclusion criteria. The study was approved by the Ethics Committee at Hiroshima University Graduate School of Biomedical and Health Sciences. All patients provided written informed consent after receiving a detailed explanation of the study, including its timeline, aims, risks and benefits of participation, and expectations regarding compliance.

### Computed tomography and radiographic superposition

**Fig. 1:**
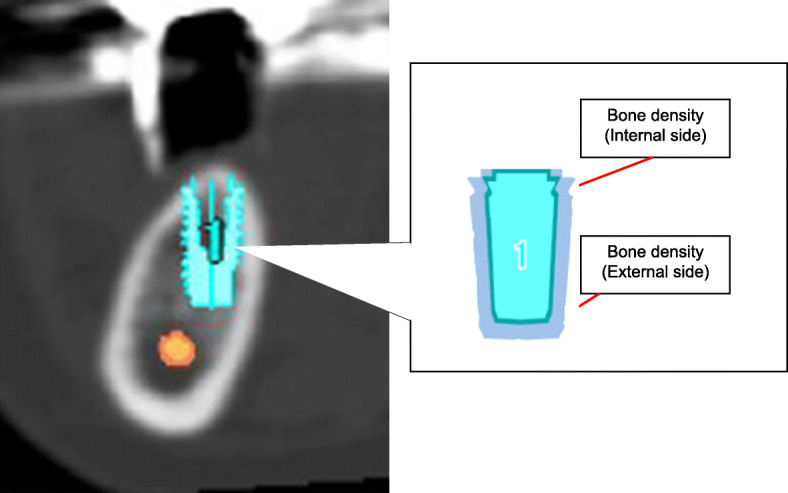
Measurements on computed tomography (CT) images. The imaging data are stored in Digital Imaging and Communications in Medicine format and loaded into an implant planning software program (Simplant®, Dentsply Implants, Leuven, Belgium). Buccopalatal or buccolingual cross-sectional CT slices are used to measure the bone density at the implant placement sites in Hounsfield units. The mean internal and external side values are used as the bone densities

Each patient underwent a spiral computed tomography (CT) scan. The data were stored in Digital Imaging and Communications in Medicine format and loaded into implant planning software (Simplant®, Dentsply Implants, Leuven, Belgium). This allowed the implant placement sites to be pinpointed on the corresponding cross-sectional CT slices. Bone density was measured in Hounsfield units on buccopalatal or buccolingual cross-sectional CT slices at the sites of implant placement. The mean bone density values for the internal and external sides were used as the cancellous bone densities (Fig. 1).

### Measurement of BTMs

BTM levels were measured in blood samples obtained simultaneously with routine blood testing before implant surgery by using ELISA. Sampling was performed in the morning in fasting subjects who had abstained from physical exercise for 24 h as recommended [16].

The BTMs selected for measurement in this study were covered by health insurance in Japan. The bone formation markers measured were osteocalcin (OC) and bone-specific alkaline phosphatase (BAP); the bone resorption markers measured were pyridinoline cross-linked carboxyterminal telopeptide of type I collagen (ICTP) and crosslinked N-telopeptide of type I collagen (NTX). The reference range for each BTM marker was set to within the established mean for healthy individuals (mean ± 1.96 SD) [17]. The normal ranges for BTM values are as follows: OC, 2.5–13.0 ng/ml; BAP, 3.7–20.9 ng/ml for men and 3.9–14.5 ng/ml in women; ICTP, < 4.5 ng/ml; and NTX, 9.5–17.7 ng/ml for men and 7.5–16.5 ng/ml for women. The abnormal group was defined as at least one BTM value outside the normal range. The blood samples were analyzed in the Division of Medical Laboratory at the Hospital.

### Surgery

Implants (SLA® (sandblasted and acid-etched, internal connection system), Straumann AG, Basel, Switzerland, or Branemark® System Mk-III (TiUnite, external connection system), Nobel Biocare, Gothenburg, Sweden) were used depending on the patient’s clinical characteristics. The operating surgeon was blinded to the bone analysis results and was unaware of the bone density measurements at the implant sites. Seventeen SLA® implants were placed in nine patients and eighteen Branemark® System Mk-III implants in nine patients. The prosthetic rehabilitation phase was started after a healing period of 2–4 weeks. Implant surgery and prosthetic rehabilitation were performed by experienced prosthodontists.

### Measurement of marginal bone loss

During the follow-up observation period, at least the final restoration was repaired and the marginal bone loss (MBL) was measured on dental radiography using Image J software (National Institutes of Health, Bethesda, MD, USA). The reference points for the measurements included the implant platform (the horizontal interface between the implant and the abutment), the implant tip, and the first bone-implant contact (FBIC). The length from the implant platform to the FBIC was defined as the marginal bone level. The marginal bone level was measured in mm using the ratio of the real implant length and the length from the implant platform to the tip on the images.

**Fig. 2:**
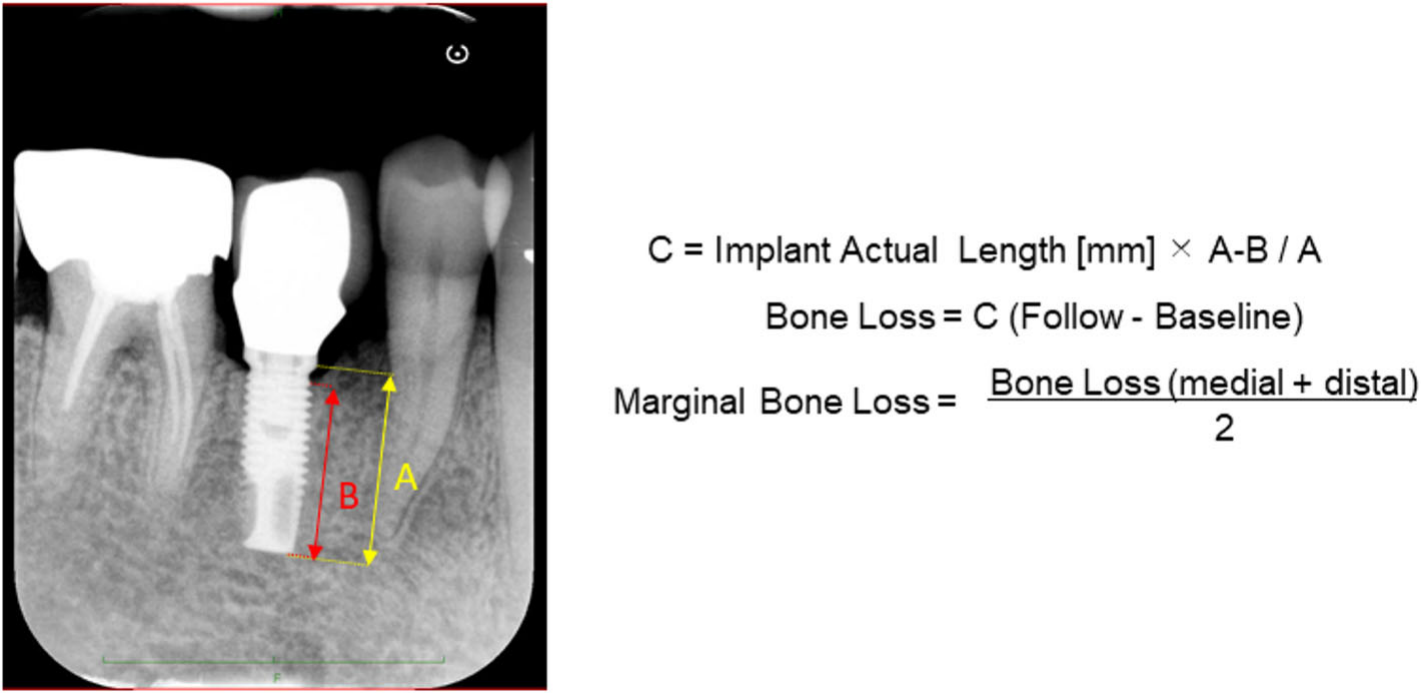
Measurement of marginal bone loss (MBL) on dental radiography. MBL is measured using Image J software (National Institutes of Health, Bethesda, MD, USA). The reference points for the measurements are the implant platform (the horizontal interface between the implant and the abutment), the implant tip, and the first bone-implant contact (FBIC). The length from the implant platform to the FBIC defines the marginal bone level. The marginal bone level is measured in mm using the ratio of the real implant length and the length from the implant platform to the tip on the images

MBL values were measured as the difference in the marginal bone level at the follow-up period and at the baseline. The mean MBL value of mesial and distal sides was used for the study. This method is diagrammatically represented in Fig. 2.

### Statistical analysis

Mann–Whitney test was used to determine the differences in the bone density values [HU] between the normal and abnormal group. A value of *P* < 0.05 was considered significant, and the effect size *r* was subsequently shown.

## Results

Eighteen patients (11 women, 7 men; mean age 60.2 years) fulfilled the inclusion criteria. There was no significant difference between men (57.3 ± 5.0 years) and women (63.1 ± 6.0 years). After measuring the BTMs and assessing bone density, 35 implants were placed (18 MK-III, 17 SLA). The mean follow-up duration was 21.4 months (Table 1), during which time follow-up observations were made by dental hygienists at 3-month intervals. There was no evidence of peri-implantitis or implant loss over the 6–48 months post-implantation.

**Table 1:** Each parameter of the 18 patients who fulfilled the inclusion criteria

Case no.	Gender	Age [years]	Bone turnover marker				Position	Implant	Bone Density [HU]	Follow-up period [m]	Marginal bone loss [mm]
			OC	BAP	ICTP	NTX					
1	M	60.2	5.8	8.1	2.7	11.4	7┐	SLA	1112.63	10	-
							6┐	SLA	815.75	10	-
2	M	51.3	4.9	9.9	3.0	12.0	6┐	SLA	746.00	6	0.32
							5┐	SLA	893.55	6	0.12
							┌5	SLA	861.52	19	0.13
							┌6	SLA	549.77	19	0.00
							┌7	SLA	415.24	19	0.07
3	M	64.3	3.5	8.7	2.4	12.9	┌6	SLA	593.45	36	0.39
4	M	54.0	4.8	11.6	2.6	-	┌7	SLA	2001.55	19	-
5	M	60.8	5.2	13.3	2.8	9.8	7┐	Mk-III	530.18	24	0.26
							6┐	Mk-III	853.68	24	0.40
							5┐	Mk-III	951.56	24	0.27
							┌6	Mk-III	563.19	24	0.88
							┌7	Mk-III	595.82	24	0.80
6	F	58.1	8.8	14.2	2.2	14.0	4┐	Mk-III	1138.26	21	1.14
							6┐	Mk-III	1012.40	21	1.09
7	M	53.4	3.3	13.3	3.0	15.4	7┐	Mk-III	1070.54	48	0.33
							6┐	Mk-III	1213.59	48	0.58
8	F	51.2	4.2	11.6	2.7	10.4	7┐	Mk-III	898.31	20	0.61
							6┐	Mk-III	782.95	20	0.94
9	M	62.5	5.2	13.1	2.4	12.8	5┐	Mk-III	761.43	6	0.18
10	F	54.3	11.0	23.0 ↑	3.0	18.2 ↑	┌6	SLA	400.65	17	0.11
							┌7	SLA	333.05	17	0.03
11	F	60.3	14.9 ↑	21.1 ↑	3.9	12.6	┌6	SLA	405.96	12	0.20
							┌7	SLA	15.77	12	0.36
12	F	67.8	4.8	19.4 ↑	3.6	12.3	┌4	SLA	1112.42	23	0.39
13	F	75.5	4.1	26.5 ↑	3.6	16.3	┌6	SLA	395.40	48	0.03
14	F	58.8	7.8	16.9 ↑	3.1	18.0 ↑	7┐	SLA	396.18	8	0.16
							5┐	SLA	502.17	8	0.02
15	F	60.6	6.1	22.2 ↑	2.9	11.3	6┐	Mk-III	314.49	24	0.32
16	F	64.5	9.4	15.4 ↑	3.5	21.1 ↑	4┐	Mk-III	620.43	29	0.80
17	F	63.3	8.1	17.5 ↑	3.0	15.5	┌6	Mk-III	734.52	30	1.07
							┌7	Mk-III	624.92	30	0.99
18	F	63.1	7.8	15.4 ↑	3.3	22.2 ↑	6┐	Mk-III	812.32	21	0.94
							5┐	Mk-III	644.81	21	1.11

The normal ranges for BTM values are as follows: OC, 2.5–13.0 ng/ml; BAP, 3.7–20.9 ng/ml for men and 3.9–14.5 ng/ml in women; ICTP, < 4.5 ng/ml; and NTX, 9.5–17.7 ng/ml for men and 7.5–16.5 ng/ml for women

*BAP* bone alkaline phosphatase, *BTM* bone turnover marker, *HU* Hounsfield units, *ICTP* pyridinoline cross-linked carboxyterminal telopeptide of type I collagen, *ISQ* implant stability quotient, *NTX* crosslinked N-telopeptide of type I collagen, *SLA* sandblasted and acid-etched, internal connection system, *OC* osteocalcin, *Mk-III* TiUnite, external connection system

**Fig. 3:**
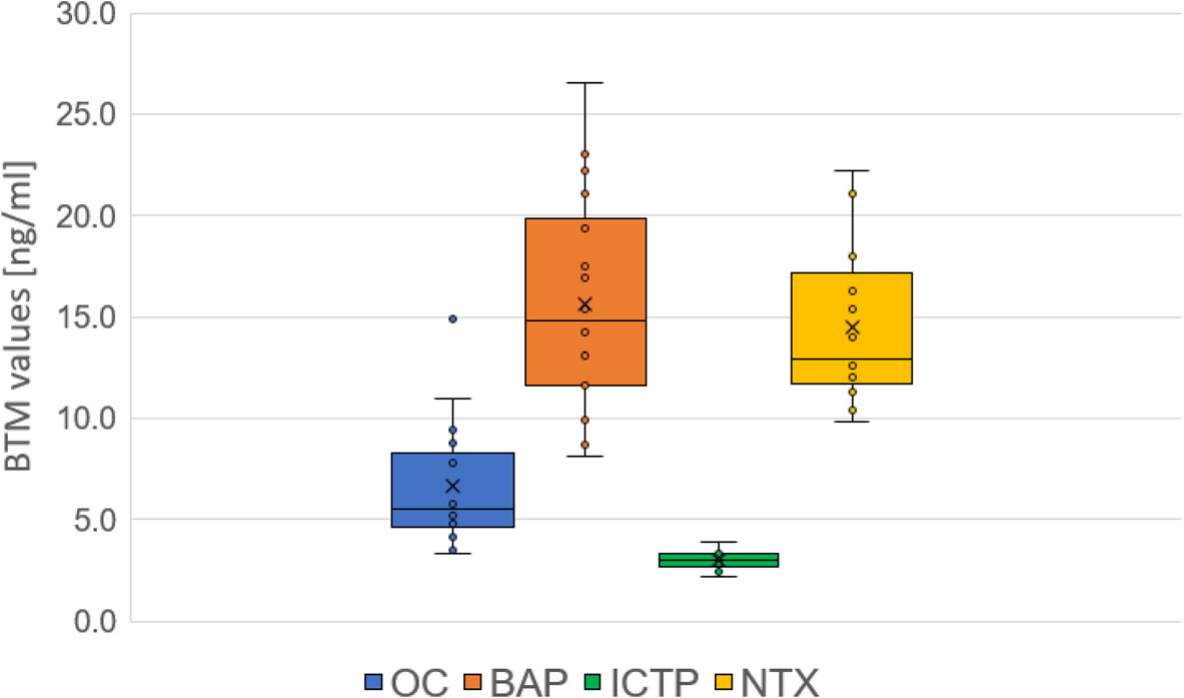
The overview on BTM values are shown. The bone turnover markers measured are osteocalcin (OC), bone-specific alkaline phosphatase (BAP), pyridinoline cross-linked carboxyterminal telopeptide of type I collagen (ICTP), and crosslinked N-telopeptide of type I collagen (NTX)

The overview on BTM values is shown in Fig. 3. The patients were divided into a normal group that had BTM values within normal limits (*n* = 9, 2 women, 7 men) and an abnormal group (*n* = 9, all women) with at least one BTM value outside the normal range. The mean age of the normal group was significantly higher than that of the abnormal group (*P* = 0.038, *r* = 0.49). The number of male patients within normal BTM values was significantly more than that of female patients (*P* = 0.0043, *r* = 0.66) (Table 2).

**Table 2:** Age, sex, and follow-up period between the normal and abnormal group

	Normal group	Abnormal group	*P* value	*r*
Age [years]	57.3	63.1	0.038*	0.49
Male (%)	7 (100)	0 (0)	0.0043*	0.66
Female (%)	2 (18)	9 (82)		
Follow-up period [months]	21.3	21.4	0.85	

*Indicate that the abnormal group has a significant difference (*p* < 0.05) compared to the normal group

**Fig. 4:**
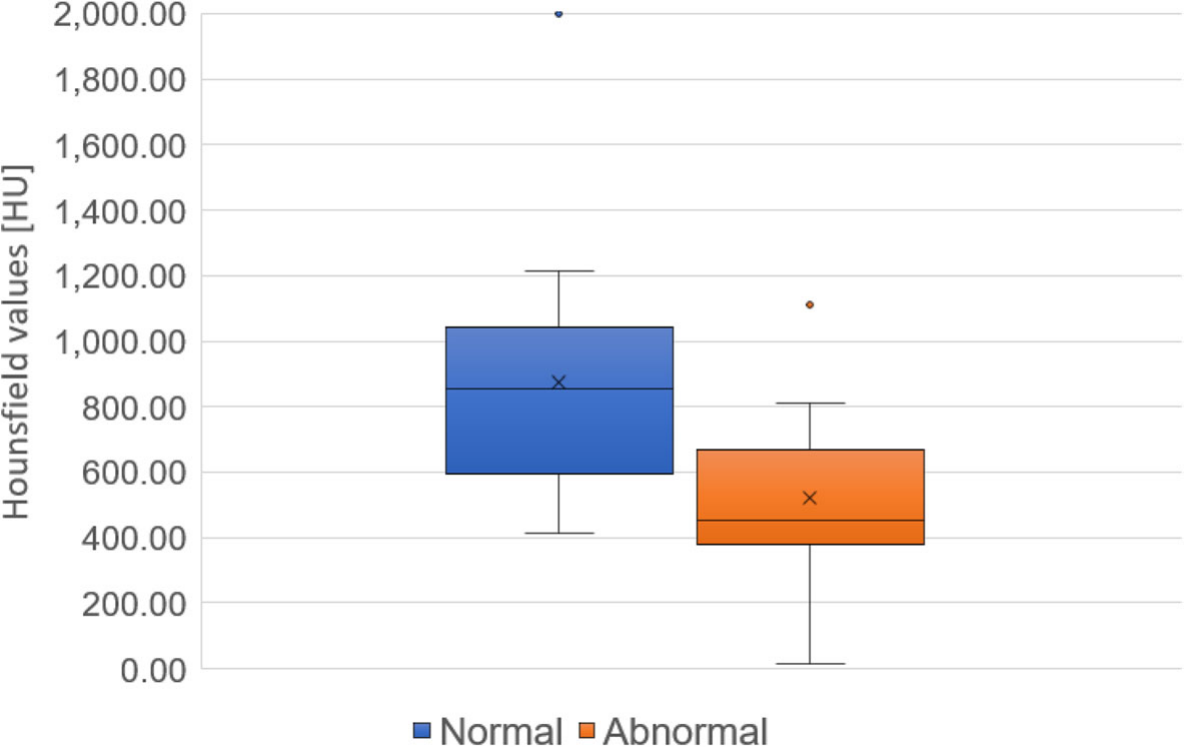
Cancellous bone densities in the normal and abnormal groups of women are shown. Cancellous bone density is significantly higher in the normal group than in the abnormal group

**Fig. 5:**
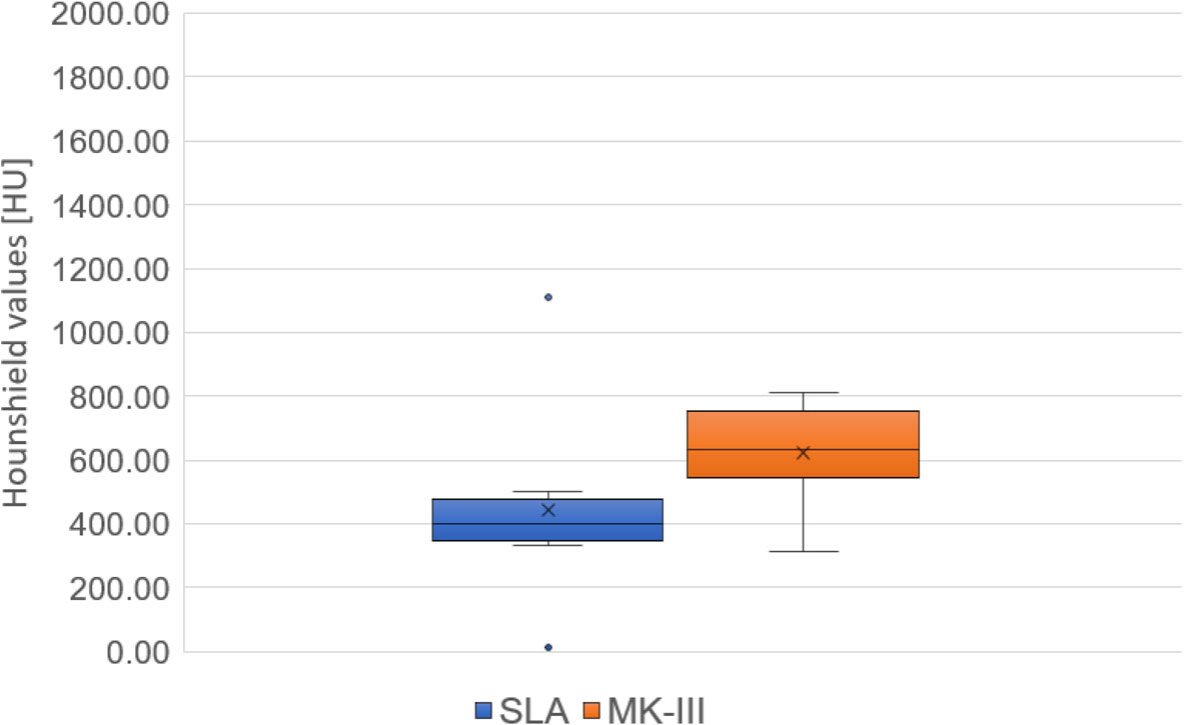
Cancellous bone densities in SLA and MK-III implants within the abnormal group. The cancellous bone density shows no significant difference between SLA and MK-III implants at baseline

The cancellous bone density is shown in Figs. 4 and 5. The cancellous bone density was significantly higher in the normal group than in the abnormal group (*P* = 0.0012, *r* = 0.55). In the abnormal group, the cancellous bone density showed no significant difference between the SLA implants and MK-III implants at baseline (*P* = 0.121, *r* = 0.41).

**Fig. 6:**
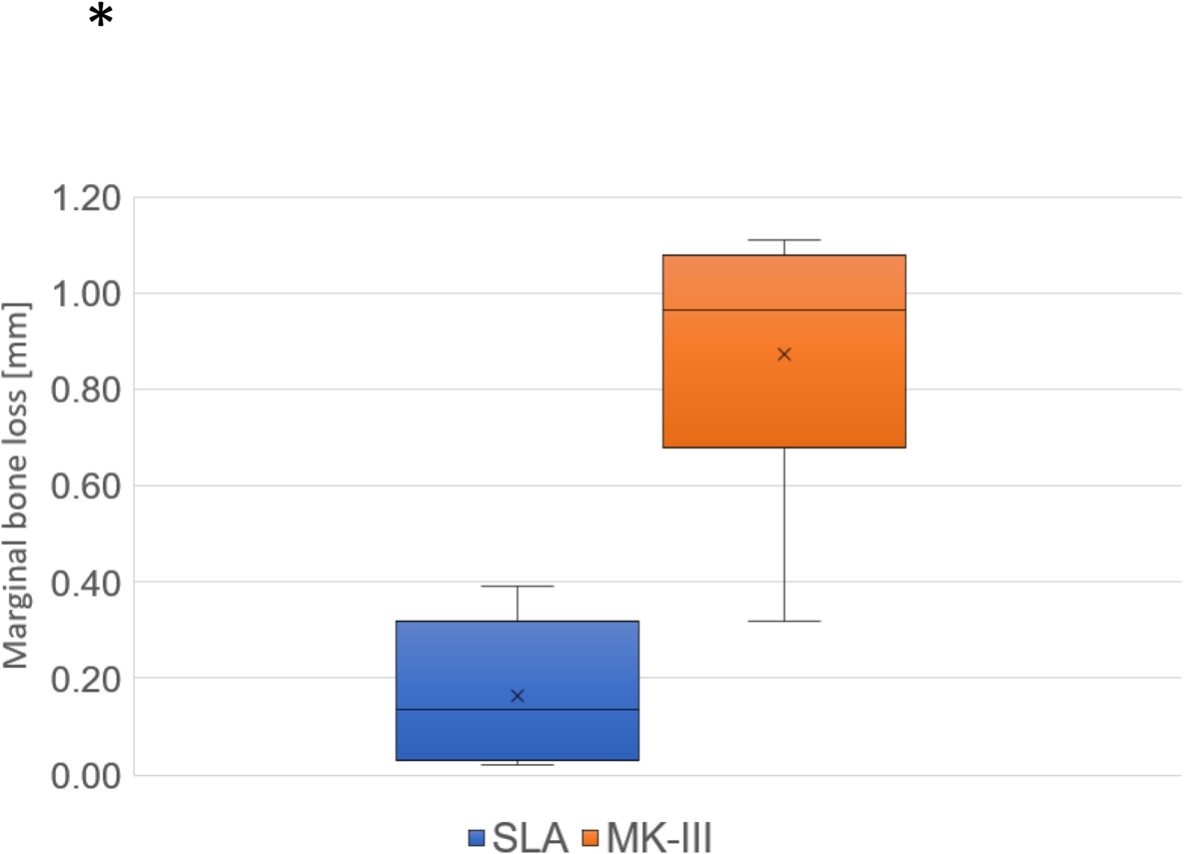
Marginal bone loss (MBL) in SLA and MK-III implants within the abnormal group. MBL is significantly larger in MK-III implants than SLA implants during the follow-up period

The MBLs are shown in Fig. 6. Among the patients who had at least one BTM measurement outside the normal range, all women, the MBL was significantly larger among those with MK-III implants than those with SLA implants in the follow-up period (*P* = 0.0045, *r* = 0.76).

## Discussion

Nine (82%) of the11 female patients in this study had at least one BTM measurement outside the normal range. The abnormal BTM values in these women were pronounced. No abnormal BTM valued were observed among the men. According to the Japanese Guidelines for the Prevention and Treatment of Osteoporosis [18], the diagnostic criteria for osteoporosis include the following: (1) fragility fractures and low BMD (i.e., nontraumatic bone fractures and a BMD < 80% of the young adult mean) in an adult or (2) a BMD < 70% of the young adult mean without fragility fractures in an adult. Furthermore, the purpose of BTM measurement is to evaluate bone metabolism in patients who have already been diagnosed with osteoporosis; it is not used for diagnostic purposes [19]. Therefore, in this study, patients with abnormal BTM measurements were not referred for consultation with an orthopedist before receiving dental implants unless they had objective or subjective symptoms of osteoporosis. According to our results, slightly greater than 80% of women in this study had high serum BTM levels and none of the male patients had abnormal BTM levels. We found that cancellous bone densities in patients with high BTMs were significantly lower than the cancellous bone densities in patients with normal BTMs. This indicates that the cancellous bone in the group with abnormal BTMs was poorer than that in the group with normal values at baseline.

According to the traditional concept of sexually dimorphic bone growth, male hormones (androgens) stimulate bone growth during puberty, whereas female hormones (estrogens) inhibit bone mineral acquisition. Moreover, men tend to lose less bone than women during the aging process because they do not experience a physiologic equivalent of menopause [20]. The results of our study are consistent with earlier reports. Biochemical BTMs reflect whole-body rates of bone resorption and formation and are likely to reflect changes in the number of bone remodeling sites [21]. Almasoud et al. reported that there was no significant difference in the cancellous bone density in the edentulous sites between the male and female patients [22]. Therefore, we mix-matched the measurements between male and female patients.

The cancellous bone density in the abnormal BTM group was significantly lower than that in the normal BTM group. This suggests that cancellous bone with a high BTM was likely to actually be poor medullary bone. There were three patterns of low BMD: high values of bone formation markers only, high values of both bone formation and bone resorption markers, and high values of bone resorption markers only. In this study, the BMDs were low even in patients who only had abnormal bone formation marker values. The BTM reflects bone turnover, and we considered that the BMD decreased because of the increased bone turnover. Given that bone is repeatedly forming and resorbing, we suggest that there were aspects of bone formation that were detected with an inclination towards bone resorption in this case. Therefore, we believe that attention should still be paid to any decline in bone quality even when it is solely the bone formation marker that is high. Biochemical BTMs provide information on bone resorption and formation, correlate with the rate of bone loss [23], and predict the likelihood of hip fracture [24]; however, we only assessed BTMs at baseline in this study. Therefore, we need to remeasure these levels when following up on these patients because accelerated bone turnover, indicated by high BTM levels, implies bone loss [25].

In the abnormal group, the cancellous bone density showed no significant difference between SLA (internal connection system) and MK-III (external connection system) at baseline. Meanwhile, MBL was significantly larger in MK-III implants than in the SLA implants during the follow-up period. Norton reported that the incorporation of an internal conical interface could dramatically enhance the ability of a dental implant unit to resist bending forces [26]. Asvanund et al. reported that there were more stresses generated at the implant-abutment connection level by external implant abutment connections than by internal implant abutment connections [27]. The load is concentrated mainly on the implant abutment interface, thus reducing the stress concentration of the internal connection implants that use the platform switching concept in the peri-implant bone region [28]. Therefore, the present study findings agree with those of the previous studies. We suggest that the MBL of external connection system implants in patients with reduced bone quality was increased by stressing the bone, even though it seems that there is no difference in BMD on the radiographic assessment.

Many reports of the relationship between dental implants and osteoporosis have been published, but few have focused on bone quality. Bone quality is multifactorial and difficult to classify because it varies from patient to patient. The present study focused on BTM, which is one of the clinical indicators of bone quality. A prospective study confirmed that elevation of a BTM can predict fracture [29]. It has also been confirmed that BTM elevation is a risk factor for fracture independent of bone density [30, 31]. Moreover, it has been reported that BTM measurements, which are estimates of systemic bone quality, are useful for evaluating periodontal disease and the condition of the jawbone [32, 33]. In the future, evaluations of bone strength in women will require assessments of both bone metabolism markers and the density of cancellous bone.

This study had a limitation; that was the imbalance in the number of the implants between male and female patients. Only two SLA implants in the normal group were placed in women, so we could not separately analyze the data according to the sex of the patients. Further research is needed with a greater sample size of women to validate these results and account for these limitations.

## Conclusion

Within the limitations of this study, we found that up to 80% of women in this study, in whom implant treatments were planned, had high serum BTM levels, and that there was no man with abnormal BTM levels. We also showed that the cancellous bone density in patients with a high BTM value was significantly lower than that in patients with a normal BTM value, indicating that the cancellous bone in the abnormal group was poorer than that in the normal group at baseline. Female patients who undergo implant treatments may have reduced bone quality; evaluations of bone strength will require assessments of both BTMs and the density of cancellous bone.

## Data Availability

The datasets used and analyzed during the current study are available from the corresponding author on reasonable request.

## Abbreviations

BTMs: Bone turnover markers
CT: Computed tomography
MBL: Marginal bone loss
BMD: Bone mineral density
